# Evaluating the accuracy of generative artificial intelligence models in dental age estimation based on the Demirjian's method

**DOI:** 10.3389/fdmed.2025.1634006

**Published:** 2025-07-29

**Authors:** Allan Abuabara, Thais Vilalba Paniagua Machado do Nascimento, Seandra Maria Trentini, Angela Mairane Costa Gonçalves, Maria Angélica Hueb de Menezes-Oliveira, Isabela Ribeiro Madalena, Svenja Beisel-Memmert, Christian Kirschneck, Livia Azeredo Alves Antunes, Cristiano Miranda de Araujo, Flares Baratto-Filho, Erika Calvano Küchler

**Affiliations:** ^1^ Post-Graduation Program in Health and Environment, University from the Joinville Region – Univille, Joinville, Brazil; ^2^ School of Dentistry, Tuiuti University of Paraná – UTP, Curitiba, Brazil; ^3^ Department of Biomaterials, University of Uberaba – UNIUBE, Uberaba, Brazil; ^4^ Department of Orthodontics, University Hospital Bonn, Medical Faculty, Bonn, Germany; ^5^ Postgraduate Program in Dentistry, Health Institute of Nova Friburgo, Fluminense Federal University, Niterói, Rio de Janeiro, Brazil

**Keywords:** artificial intelligence, generative artificial intelligence, clinical decision-making, large language models, evidence-based dentistry, age determination by teeth

## Abstract

**Introduction:**

Dental age estimation plays a key role in forensic identification, clinical diagnosis, treatment planning, and prognosis in fields such as pediatric dentistry and orthodontics. Large language models (LLM) are increasingly being recognized for their potential applications in Dentistry. This study aimed to compare the performance of currently available generative artificial intelligence LLM technologies in estimating dental age using the Demirjian's scores.

**Methods:**

Panoramic radiographs were analyzed using Demirjian's method (1973), with each left permanent mandibular tooth classified from stage A to H. Untrained LLM, ChatGPT (GPT-4-turbo), Gemini 2.0 Flash, and DeepSeek-V3 were tasked with estimating dental age based on the patient's Demirjian score for each tooth. Due to the probabilistic nature of ChatGPT, Gemini, and DeepSeek, which can produce varying responses to the same question, three responses were collected per case per day (three different computers) from each model on three separate days. The age estimates obtained from LLM were compared to the individuals’ chronological ages. Intra- and inter-examiner reliability was assessed using the Intraclass Correlation Coefficient (ICC). Model performance was evaluated using Mean Absolute Error (MAE), Root Mean Squared Error (RMSE), Coefficient of Determination (*R*^2^), and Bias.

**Results:**

Thirty panoramic radiographs (40% female, 60% male; mean age 10.4 ± 2.32 years) were included. Both intra- and inter-examiner ICC values exceeded 0.85. ChatGPT and DeepSeek exhibited comparable but suboptimal performance, with higher errors (MAE: 1.98–2.05 years; RMSE: 2.33–2.35 years), negative *R*^2^ values (−0.069 to −0.049), and substantial overestimation biases (1.90–1.91 years), indicating poor model fit and systematic flaws. Gemini demonstrated intermediate results, with a moderate MAE (1.57 years) and RMSE (1.81 years), a positive *R*^2^ (0.367), and a lower bias (1.32 years).

**Discussion:**

This study demonstrated that, although LLM like ChatGPT, Gemini, and DeepSeek can estimate dental age using Demirjian's scores, their performance remains inferior to the traditional method. Among them, DeepSeek-V3 showed the best results, but all models require task-specific training and validation before clinical application.

## Introduction

Dental age estimation, or determining age through the examination of teeth, is widely used in archaeology, anthropology, medicine, and both clinical and forensic dentistry to assess an individual's age. Age estimation is fundamental for diagnosing developmental disorders and planning orthodontic interventions (1, 2). Various methods have been developed for dental age estimation, with the majority relying on radiographic or tomographic imaging (3). One of the most widely used and extensively studied methods, primarily applicable to children and adolescents, is Demirjian's method (4), which categorizes the development of seven left mandibular permanent teeth into eight stages (A–H).

Large Language Models (LLM) are a form of Artificial Intelligence (AI) designed to replicate human language comprehension and generation. Examples of LLM include the GPT series (OpenAI), Gemini (Google), and DeepSeek (High-Flyer). By analyzing patterns and relationships within large datasets, LLM can predict the most probable words or phrases to follow in a given context. These models are increasingly recognized for their potential applications in science, particularly in the medical field and dentistry, where they have been explored for various tasks, such as enhancing diagnostic accuracy (5–7). A recent scoping review (5) demonstrated that these technologies might enhance dental care, especially in orthodontic settings. The findings highlighted a growing global interest in the application of LLM in orthodontics, suggesting increasing acceptance within the field.

The growing use of LLM across various fields, including dentistry, raises concerns about their accuracy and reliability. Although studies highlight the promising potential of artificial intelligence tools in supporting the implementation of evidence-based dentistry, their current limitations may lead to potentially harmful healthcare decisions if not used with caution (2, 7). LLM can also produce irrelevant information, vague responses, or content that is not entirely accurate (7). Most studies evaluate LLM after they have undergone training. However, no information was found regarding how these models perform without any prior training, particularly in dental age estimation.

DeepSeek, ChatGPT, and Google Gemini are currently trending LLM technologies for reasoning, multimodal capabilities, and general linguistic performance worldwide. The potential applications of certain LLM in dentistry have yet to be fully explored. This study aimed to compare the performance of currently available untrained generative artificial intelligence technologies, specifically LLM, in estimating dental age using Demirjian's dental development stages.

## Materials and methods

### Study design

This study employed a comparative design, using simulated scenarios generated from real data to evaluate the performance of different LLM-based artificial intelligence models in dental age estimation. Simulations based on real-world data allowed for controlled, reproducible, and ethical evaluation of model performance while preserving the complexity of clinical scenarios.

### Data source and sample selection

This study utilized secondary data from digital panoramic radiographs collected between 2018 and 2023. Sampling was performed by convenience, selecting images from an existing database of panoramic radiographs originally acquired for dental treatment purposes. Panoramic radiographs of healthy individuals of both sexes were included. Only individuals between 3 and 16 years old were considered eligible, as recommended by Demirjian's method (4). Radiographs with distortions, visible pathological alterations, or anomalies in the number or shape of teeth were excluded.

Additionally, individuals with bilateral agenesis of mandibular teeth were excluded from the study, as Demirjian's method recommends assessing the permanent mandibular teeth on the left side, excluding the third molar. In cases of unilateral tooth agenesis, the corresponding tooth on the right side was used for analysis. Patient data were anonymized. Patients' dates of birth and chronological ages were anonymized for the evaluators who tested the models. The study was approved by the Ethics Committee and conducted by the principles of the Declaration of Helsinki.

### Sample size

To determine the appropriate sample size, the correlation between the outcomes of different LLM and the Demirjian's method was initially calculated. A conservative approach was adopted by using the lowest observed correlation coefficient (*r* = 0.504) to estimate the required sample size. Considering a two-tailed test with a 5% significance level (*α* = 0.05) and 80% statistical power (*β* = 0.20), the estimated sample size was 29 evaluations per model.

### Reference method: Demirjian dental age estimation

Two trained dentists conducted the assessments using Demirjian's dental stage classification. A professor provided training with a PhD with over 20 years of experience in the field. To ensure reliability, the examiners initially analyzed 30 radiographs, resolving discrepancies through discussion with the professor until a consensus was reached. After 15 days, they independently assessed an additional 35 radiographs, followed by a third round of analysis 30 days later. The Kappa statistic was used to assess intra- and inter-examiner agreement. Statistical analysis was performed using Jamovi software (Version 2.3.26.0, https://www.jamovi.org). The inter-examiner Kappa values for the first and second rounds of analysis were 0.782 and 0.934, respectively, indicating good agreement (*κ* > 0.75). The intra-examiner agreement yielded Kappa values of 1.00 for both examiners. Each permanent mandibular tooth (from the central incisor to the second molar) on the left side was classified into one of eight developmental stages (A–H) according to Demirjian's method (4). A third researcher then applied the corresponding maturity scores from the original method to estimate dental age based on established conversion tables. In cases of tooth agenesis, the corresponding tooth on the right side was used for classification. Examiners performed the evaluations in a blinded manner, without access to the individual's age or sex.

### Prompt design and LLM

The ChatGPT model GPT-4-turbo (OpenAI), Gemini 2.0 Flash (Google Inc.), and DeepSeek-V3 (High-Flyer), all of which were offered free, were used in this study. The following question was applied using the dental maturity scores of Demirjian's method (A–H) and the gender of each patient: “*Provide the estimated dental age based on the Demirjian's method according to the data below (with one decimal place): Sex: male, Central incisor: D, Lateral incisor: D, Canine: E, First premolar: F, Second premolar: E, First molar: G, Second molar: F.*” The tests were conducted on March 31, April 3, and April 5, 2025.

To ensure methodological consistency and reproducibility, all prompts submitted to the LLM were standardized across experiments. Each model received identical input phrasing and structure, minimizing variability due to prompt design and allowing for a fair comparison of performance. By controlling this variable, we aimed to isolate model-specific behavior and ensure that differences in responses were attributable to the models themselves rather than inconsistencies in the experimental setup.

Due to the probabilistic nature of LLM, which may generate different responses to the same prompt, each scenario was tested on three different computers, with three evaluations conducted per day over three consecutive days (8, 9). This protocol resulted in a total of 27 evaluations per scenario, comprising nine tests per scenario for each of the three LLM. Intra- and inter-examiner reliability was assessed using the Intraclass Correlation Coefficient (ICC).

### Outcome measures

As outcome measures, the responses generated by the LLM and the participants' chronological age were considered. Chronological age was determined by subtracting the radiographic date from the individual's birth date (in days) and dividing the result by 365.25, accounting for leap years.

### Statistical analysis

Residual errors, defined as the difference between predicted age from LLM and chronological age, were calculated for each model and time point. These errors formed the basis of all subsequent analyses. To assess the performance of the predictive models, four statistical metrics were calculated: Mean Absolute Error (MAE), Root Mean Squared Error (RMSE), Coefficient of Determination (*R*^2^), and Bias (mean error). These metrics were selected to provide a comprehensive evaluation of the models in terms of accuracy and systematic error.

• MAE represents the average absolute difference between the predicted and actual values, indicating the average magnitude of the prediction errors.
• RMSE provides additional insight by penalizing larger errors more heavily, making it particularly useful for detecting substantial discrepancies.
• *R*^2^ quantifies the proportion of variance in the actual values that is explained by the model, serving as an indicator of goodness-of-fit.
• Bias reflects the average deviation of predicted values from actual values, allowing for the identification of systematic overestimation or underestimation tendencies.

**Figure 1:**
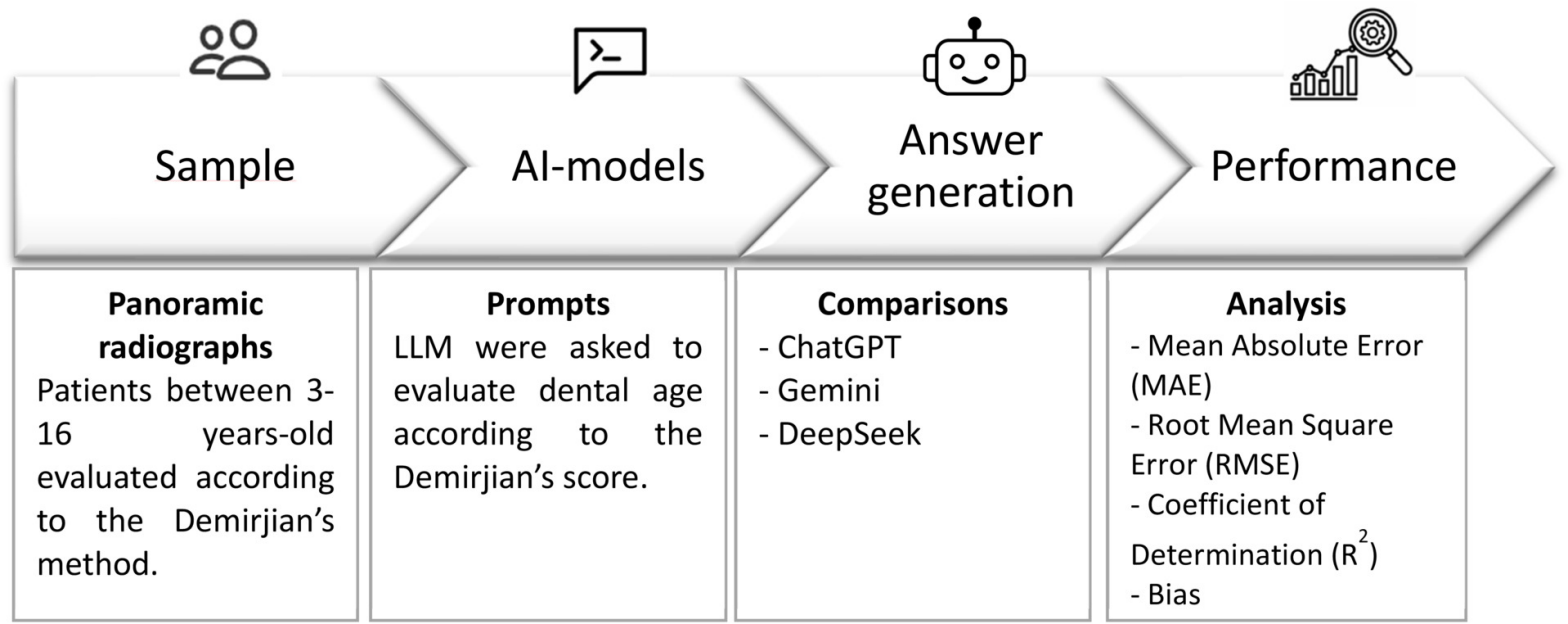
Workflow for evaluating large language models (LLM) in dental age estimation using the Demirjian's method. Models were prompted and compared (ChatGPT, Gemini, DeepSeek), with performance assessed by Mean Absolute Error (MAE), Root Mean Square Error (RMSE), Coefficient of Determination (*R*^2^), and bias. AI: Artificial Intelligence.

Comparisons between predictive models were conducted separately for each time point using repeated measures ANOVA (ANOVA-RM), considering the same individual assessed by all three models. When the ANOVA indicated statistical significance (*p* < 0.05), *post-hoc* pairwise comparisons with Holm correction were performed to adjust for multiple testing. Similarly, each model underwent a repeated measures ANOVA to investigate possible differences across time points (day 1 = T1, day 2 = T2, and day 3 = T3). When significance was found, *post-hoc* pairwise comparisons with the Holm correction were also applied. All analyses were performed in Python (version 3.10.12) using the Pandas, Numpy, Scipy, Statsmodels, and Scikit-Learn libraries. Figure 1 shows the sequence of the main steps of the study.

## Results

### Sample characterization

A total of 30 panoramic radiographs were included and analyzed, 40% from females and 60% from males. The mean age was 10.4 ± 2.32 years for both sexes, with an age range of 7–14.9 years.

### Reliability of reference assessments

The intra- and inter-examiner ICC values were both greater than 0.85, indicating excellent agreement. The data supporting the findings of this study are available at https://doi.org/10.6084/m9.figshare.29045501.v1.

### Performance of LLM vs. reference

The analysis revealed significant disparities in performance among the evaluated methods (Table 1). The Demirjian method, when performed traditionally, had a MAE (1.21 years) and RMSE (1.62 years) alongside a clinically acceptable *R*^2^ (0.497) and minimal bias (0.14 years). ChatGPT and DeepSeek exhibited comparable but suboptimal performance, with higher errors (MAE: 1.98–2.05 years; RMSE: 2.33–2.35 years), negative *R*^2^ values (−0.069 to −0.049), and substantial overestimation biases (1.90–1.91 years), indicating poor model fit and systematic flaws. Gemini demonstrated intermediate results, with a moderate MAE (1.57 years) and RMSE (1.81 years). However, its positive *R*^2^ (0.367) and lower bias (1.32 years) suggest partial utility despite persistent overestimation.

**Table 1:** Performance metrics of large language models (LLM) compared to the traditional demirjian's method across three evaluation days (T1, T2, T3).

Method	MAE T1	RMS T1	*R*^2^ T1	Bias T1	MAE T2	RMSE T2	*R*^2^ T2	Bias T2	MAE T3	RMSE T3	*R*^2^ T3	Bias T3
Demirjian	1.21	1.62	0.497	0.14	—	—	—	—	—	—	—	—
ChatGPT	1.98	2.35	−0.069	1.91	2.08	2.45	−0.16	2.03	1.88	2.24	0.035	1.77
Gemini	2.05	2.33	−0.049	1.9	2.17	2.47	−0.178	2.07	2.8	3.29	−1.085	2.67
DeepSeek	1.57	1.81	0.367	1.32	1.88	2.21	0.056	1.71	1.76	2.03	0.204	1.57

MAE, mean absolute error; RMSE, root mean square error; *R*^2^, coefficient of determination. Lower values indicate better performance for MAE, RMSE, and absolute Bias. *R*^2^ closer to 1 represents a better model fit.

Demirjian: Demirjian's method performed traditionally.

T1, T2, T3: day 1, day 2, and day 3.

Table 2 presents a comparison of the MAE values for the three LLM across the three days (T), reporting mean errors with standard deviations and corresponding ANOVA *p*-values. Results indicate that DeepSeek consistently achieved the lowest mean errors across all days (T1: 1.32 ± 1.26; T2: 1.71 ± 1.43; T3: 1.57 ± 1.31), demonstrating superior accuracy to ChatGPT and Gemini. Gemini exhibited the highest variability, particularly on T3 (2.67 ± 1.95). All ANOVA *p*-values were statistically significant (*p* < 0.05), with T3 showing the most pronounced differences (*p* = 0.0002).

**Table 2:** Mean absolute error (MAE) values (mean ± SD) of large language models (LLM) across evaluation periods (T1, T2, T3).

Day (T)	Chat PT (mean ± SD)	Gemini (mean ± SD)	DeepSeek (mean ± SD)	*p*-value (0.05)
T1	1.91 ± 1.40	1.90 ± 1.38	1.32 ± 1.26	0.0018
T2	2.03 ± 1.40	2.07 ± 1.37	1.71 ± 1.43	0.0442
T3	1.77 ± 1.39	2.67 ± 1.95	1.57 ± 1.31	0.0002

Anova was used.

T1, T2, T3: day 1, day 2, and day 3.

SD, standard deviation.

**Figure 2:**
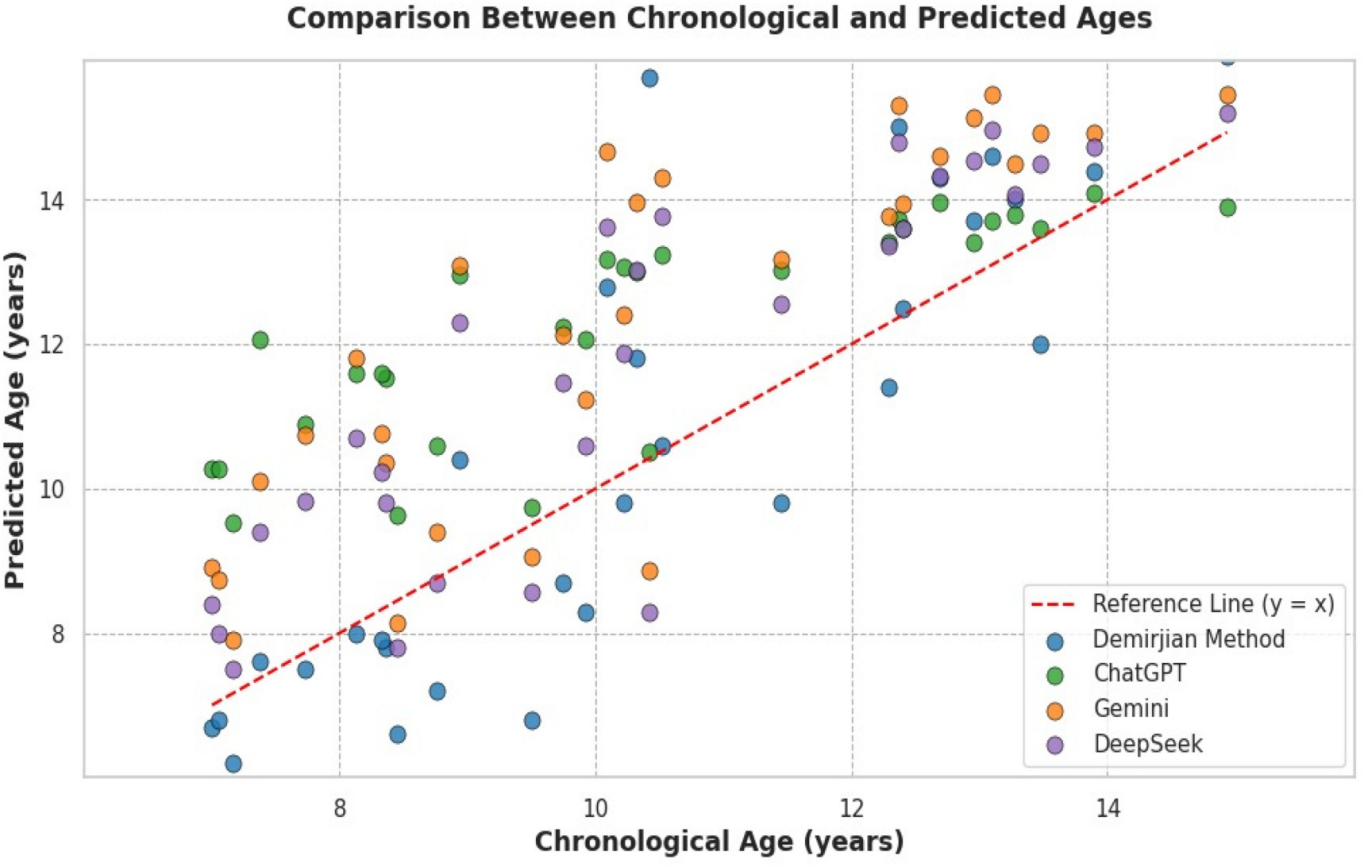
Scatter plot comparing chronological age and predicted dental age estimated by the Demirjian's method and three LLM (ChatGPT, Gemini, and DeepSeek). The red dashed line represents the ideal prediction (*y* = *x*).

Table 3 presents a comparison of results across different time points for each LLM, with statistical significance confirmed by ANOVA (*p* < 0.001 for all models). The performance analysis of the models over the three testing periods revealed statistically significant differences for all models (*p* < 0.001). However, when considering the magnitude of variations in absolute errors, ChatGPT exhibited only slight fluctuations (T1: 1.91 ± 1.40; T2: 2.03 ± 1.40; T3: 1.77 ± 1.39), indicating relative stability. The DeepSeek model also demonstrated consistency, with the lowest mean error values (T1: 1.32 ± 1.26; T2: 1.71 ± 1.43; T3: 1.57 ± 1.31), despite the statistical differences. In contrast, the Gemini model showed a progressive increase in errors, with poorer performance on the third day (T1: 1.90 ± 1.38; T2: 2.07 ± 1.37; T3: 2.67 ± 1.95), suggesting instability in predictions over time. Figure 2 shows the comparative analysis of predicted and chronological.

**Table 3:** Comparison of mean absolute error (MAE) values (mean ± SD) across different evaluation days (T1, T2, T3) for each large language models (LLM).

Method	T1 (mean ± SD)	T2 (mean ± SD)	T3 (mean ± SD)	*p*-value
ChatGPT	1.91 ± 1.40	2.03 ± 1.40	1.77 ± 1.39	<0.001
Gemini	1.90 ± 1.38	2.07 ± 1.37	2.67 ± 1.95	<0.001
DeepSeek	1.32 ± 1.26	1.71 ± 1.43	1.57 ± 1.31	<0.001

Anova was used.

T1, T2, T3: day 1, day 2, and day 3.

SD, standard deviation.

## Discussion

LLM are trained on vast datasets sourced from the internet, and the training process involves exposing them to extensive and diverse datasets, encompassing a broad range of text and, increasingly, other modalities such as code and images. Previous research has explored the utility and performance of LLM across various educational domains, including medicine and healthcare, computer and data science, law, business, journalism, media, and language learning (10). Although findings have been mixed, particularly when comparing ChatGPT's performance on standardized exams to that of human students, these studies highlight both the potential and the limitations of such models in academic contexts.

The growing use of LLM across various fields, including dentistry, raises concerns about their accuracy and reliability. The recent wave of generative artificial intelligence chatbots, theoretically capable of instantly generating evidence-based responses to scientific queries and thus acting as the dentist's “chairside personal scientific consultant,” appears to have the potential to be an ideal tool for the successful implementation and enhancement of evidence-based dentistry. However, as demonstrated in the results of this study, their use must be approached with extreme caution. While these chatbots may resemble a scientific consultant, the accuracy and reliability of their responses require careful evaluation before being fully integrated into clinical practice.

Demirjian's method (4) relies on a table of values, where each letter (A–H) assigned to each evaluated tooth corresponds to a specific value based on sex. These values are then summed, and the estimated dental age is determined by referencing a second table. Similar to the function of a calculator, the results were expected to be consistent and accurate; however, this was not observed. Due to the probabilistic architecture of ChatGPT, Gemini, and DeepSeek, which allows for variability in responses to identical prompts, the LLM yielded different outputs (8, 9). DeepSeek outperformed ChatGPT and Gemini in reliability and accuracy. Gemini showed the highest variability, while ChatGPT remained relatively stable but less accurate than DeepSeek. These tools should undergo further training and be subjected to new tests to assess their reliability and accuracy.

While several machine learning approaches have demonstrated high accuracy in dental age estimation using structured inputs (2), this study deliberately focused on the performance of untrained LLMs due to their increasing availability and adoption in clinical and academic settings. Unlike traditional models that require structured datasets and explicit training processes, LLM are designed to interpret and generate text-based responses from natural language prompts, offering a more flexible and accessible interface for clinicians and students. The rationale for selecting ChatGPT, Gemini, and DeepSeek-V3 lies in their widespread usage, multimodal capabilities, and claimed ability to perform reasoning tasks across various domains, including medicine and dentistry (11). This study sought to explore whether such models, in their freely accessible and untrained form, could accurately perform a structured task that traditionally relies on domain-specific knowledge, such as Demirjian's method. Therefore, the goal was not to compare these models against traditional machine learning regressors, but rather to critically evaluate their current limitations and potential when used “as-is” by professionals in real-world scenarios.

Machine learning algorithms have demonstrated greater accuracy in estimating dental age compared to traditional methods (2, 12–14). However, the use of the free version of these models, without any form of machine learning training, proved to be ineffective, yielding inconsistent and erroneous results across all the tested types. Professionals and students must be attentive when using these tools. One aspect involves utilizing them to generate effective and accurate results after proper training, while another completely different consideration is the use of free versions. The Dental Age app (Crescendo Treinamentos Avançados Ltda, Curitiba, Brazil), available on the Apple App Store and Google Play Store, is a technological tool designed to assist in assessing dental age. The Dental Age app replicates the method proposed by Demirjian et al. (4), which begins with the identification of the patient's sex, followed by the assignment of scores to each analyzed tooth (A–H) (15). Upon completing the analysis, the application automatically calculates the dental age. The app functions as a calculator and may serve as an alternative solution, while artificial intelligence-based methods are not yet available to professionals.

Other applications of artificial intelligence in orthodontics include the measurements of cephalometric radiographs (16). The artificial intelligence used alone proved to be not accurate enough for landmark identification and, accordingly, not precise in the generation of lateral cephalometric measurements; however, it can serve as an auxiliary tool.

A relevant limitation of this study lies in the use of a single prompt to evaluate the performance of LLM. The standardization of the instruction was a deliberate methodological choice aimed at ensuring experimental consistency and controlling for prompt-related variability. This approach allowed us to isolate the intrinsic behavior of each model, avoiding biases arising from linguistic variations in the input. However, it is well known that LLM are highly sensitive to the structure, terminology, and phrasing of prompts, which can significantly influence the responses they generate. Nonetheless, relying solely on multiple prompt variations may not effectively address the core limitation, as it does not overcome the fundamental issue of lacking task-specific training for structured clinical applications.

In this regard, future studies should move toward integrating LLM with supervised deep learning techniques, enabling fine-tuning based on domain-specific datasets from dentistry. Customizing LLM for tasks such as dental age estimation, particularly when using standardized classification systems like Demirjian's method, may substantially enhance accuracy, stability, and consistency of outputs. Moreover, hybrid strategies that combine the linguistic flexibility of LLM with the numerical precision of traditional regression-based algorithms could represent a promising direction for safe and effective clinical applications.

Studies indicate that despite uncertainties surrounding the ethical use of artificial intelligence, a significant number of students express their intention to continue using LLM for academic purposes. As a result, it is essential to develop programs that enhance students' understanding of artificial intelligence chatbot technologies (10, 17). These programs should focus on educating students about the capabilities, limitations, and ethical considerations associated with using such tools in academic contexts (17). This could include seminars, workshops, or courses designed to promote artificial intelligence literacy (17).

## Conclusion

This study demonstrated that, although LLM such as ChatGPT, Gemini, and DeepSeek are capable of providing dental age estimates based on Demirjian's method classifications, their performance still falls short of that of the traditional approach conducted by human examiners. Among the LLM tested, DeepSeek-V3 showed the lowest mean errors and the most excellent stability over time, whereas Gemini exhibited higher variability and a decline in performance. ChatGPT presented intermediate results with relative stability but lower accuracy.

These findings highlight that LLM not trained explicitly for this task exhibit notable limitations, including a tendency toward overestimation and inconsistent performance. While promising, these models should be used with caution and should not replace validated methods until they undergo supervised training and robust clinical validation. The indiscriminate use of such tools may lead to incorrect interpretations with potential clinical and legal implications. Future studies should explore fine-tuning these models and evaluate their performance across different population contexts before considering practical implementation.

## Data Availability

The raw data supporting the conclusions of this article will be made available by the authors, without undue reservation.

## References

[1] Kirschneck C Proff P. Age assessment in orthodontics and general dentistry. Quintessence Int. (2018) 49(4):313–23. 10.3290/j.qi.a39960 29532818

[2] Shen S Liu Z Wang J Fan L Ji F Tao J. Machine learning assisted Cameriere method for dental age estimation. BMC Oral Health. (2021) 21(1):641. 10.1186/s12903-021-01996-0 34911516 PMC8672533

[3] Cortés MM P Rojo R Alía García E Mourelle Martínez MR. Accuracy assessment of dental age estimation with the Willems, Demirjian and Nolla methods in Spanish children: comparative cross-sectional study. BMC Pediatr. (2020) 20(1):361. 10.1186/s12887-020-02247-x 32736612 PMC7393889

[4] Demirjian A Goldstein H Tanner JM. A new system of dental age assessment. Hum Biol. (1973) 45(2):211–27. 4714564

[5] Zheng J Ding X Pu JJ Chung SM Ai QYH Hung KF Unlocking the potentials of large language models in orthodontics: a scoping review. Bioengineering (Basel. (2024) 11(11):1145. 10.3390/bioengineering11111145 39593805 PMC11591942

[6] Albalawi F Khanagar SB Iyer K Alhazmi N Alayyash A Alhazmi AS Evaluating the performance of artificial intelligence-based large language models in orthodontics—a systematic review and meta-analysis. Appl Sci. (2025) 15(2):893. 10.3390/app15020893

[7] Giannakopoulos K Kavadella A Aaqel Salim A Stamatopoulos V Kaklamanos EG. Evaluation of the performance of generative AI large language models ChatGPT, Google Bard, and Microsoft Bing Chat in supporting evidence-based dentistry: comparative mixed methods study. J Med Internet Res. (2023) 25:e51580. 10.2196/51580 38009003 PMC10784979

[8] Suárez A Jiménez J Llorente de Pedro M Andreu-Vázquez C Díaz-Flores García V Gómez Sánchez M Beyond the Scalpel: assessing ChatGPT’s potential as an auxiliary intelligent virtual assistant in oral surgery. Comput Struct Biotechnol J. (2023) 24:46–52. 10.1016/j.csbj.2023.11.058 38162955 PMC10755495

[9] Xie Y Seth I Hunter-Smith DJ Rozen WM Ross R Lee M. Aesthetic surgery advice and counseling from artificial intelligence: a rhinoplasty consultation with ChatGPT. Aesthetic Plast Surg. (2023) 47(5):1985–93. 10.1007/s00266-023-03338-7 37095384 PMC10581928

[10] Ibrahim H Liu F Asim R Battu B Benabderrahmane S Alhafni B Perception, performance, and detectability of conversational artificial intelligence across 32 university courses. Sci Rep. (2023) 13(1):12187. 10.1038/s41598-023-38964-3. Erratum in: *Sci Rep*. (2023) 13(1):17101. doi: 10.1038/s41598-023-43998-8. 37620342 PMC10449897

[11] Farhadi Nia M Ahmadi M Irankhah E. Transforming dental diagnostics with artificial intelligence: advanced integration of ChatGPT and large language models for patient care. Front Dent Med. (2025) 5:1456208. 10.3389/fdmed.2024.1456208 39917691 PMC11797834

[12] Bunyarit SS Nambiar P Naidu M Asif MK Poh RYY. Dental age estimation of Malaysian Indian children and adolescents: applicability of Chaillet and Demirjian’s modified method using artificial neural network. Ann Hum Biol. (2022) 49(3-4):192–9. 10.1080/03014460.2022.2105396 35997704

[13] Galibourg A Cussat-Blanc S Dumoncel J Telmon N Monsarrat P Maret D. Comparison of different machine learning approaches to predict dental age using Demirjian’s staging approach. Int J Legal Med. (2021) 135(2):665–75. 10.1007/s00414-020-02489-5 33410925

[14] Vila-Blanco N Varas-Quintana P Tomás I Carreira MJ. A systematic overview of dental methods for age assessment in living individuals: from traditional to artificial intelligence-based approaches. Int J Legal Med. (2023) 137(4):1117–46. 10.1007/s00414-023-02960-z 37055627 PMC10247592

[15] Madalena IR Küchler EC Reis CLB Matsumoto MAN Stuani MBS Vilalba Paniagua Machado do Nascimento T Association of PTH and vitamin D-related genes with dental development in Brazilian children: a cross-sectional study. Braz Oral Res. (2025) 39:e033. 10.1590/1807-3107bor-2025.vol39.033 40172435 PMC11970514

[16] El-Dawlatly M Attia KH Abdelghaffar AY Mostafa YA Abd El-Ghafour M. Preciseness of artificial intelligence for lateral cephalometric measurements. J Orofac Orthop. (2024) 85(Suppl 1):27–33. 10.1007/s00056-023-00459-1 36894679 PMC11126516

[17] George Pallivathukal R Kyaw Soe HH Donald PM Samson RS Hj Ismail AR. ChatGPT for academic purposes: survey among undergraduate healthcare students in Malaysia. Cureus. (2024) 16(1):e53032. 10.7759/cureus.53032 38410331 PMC10895383

